# EM703 improves bleomycin-induced pulmonary fibrosis in mice by the inhibition of TGF-β signaling in lung fibroblasts

**DOI:** 10.1186/1465-9921-7-16

**Published:** 2006-01-27

**Authors:** Ying Ji Li, Arata Azuma, Jiro Usuki, Shinji Abe, Kuniko Matsuda, Toshiaki Sunazuka, Takako Shimizu, Yukiyo Hirata, Hirofumi Inagaki, Tomoyuki Kawada, Satoru Takahashi, Shoji Kudoh, Satoshi Omura

**Affiliations:** 1 Fourth Department of Internal Medicine, Nippon Medical School, Tokyo, JAPAN; 2 Department of Hygiene and Public Health, Nippon Medical School, Tokyo, JAPAN; 3 Institute of Basic Medical Sciences, University of Tsukuba, Ibaragi, JAPAN; 4 Kitasato Institute for Life Sciences, Kitasato University, Tokyo, JAPAN

## Abstract

**Background:**

Fourteen-membered ring macrolides have been effective in reducing chronic airway inflammation and also preventing lung injury and fibrosis in bleomycin-challenged mice via anti-inflammatory effects. EM703 is a new derivative of erythromycin (EM) without the bactericidal effects. We investigated the anti-inflammatory and antifibrotic effects of EM703 in an experimental model of bleomycin-induced lung injury and subsequent fibrosis in mice.

**Methods:**

Seven-week-old male ICR mice were used. All experiments used eight mice/group, unless otherwise noted in the figure legends. Bleomycin was administered intravenously to the mice on day 0. EM703 was orally administered daily to mice. All groups were examined for cell populations in the bronchoalveolar lavage (BAL) fluid and for induction of messenger RNA (mRNA) of Smad3 and Smad4 in the lung tissues by reverse transcriptase (RT)-polymerase chainreaction (PCR) on day 7. Fibroblastic foci were assessed histologically, and the hydroxyproline content was chemically determined in the lung tissues on day 28. We performed assay of proliferation and soluble collagen production, and examined the induction of mRNA of Smad3 and Smad4 by RT-PCR in murine lung fibroblast cell line MLg2908. We also examined Smad3, Smad4 and phosphorylated Smad2/3 (p-Smad2/3) protein assay by western blotting in MLg2908.

**Results:**

Bleomycin-induced lung fibrosis, and the infiltration of macrophages and neutrophils into the airspace were inhibited by EM703. The expression of Smad3 and Smad4 mRNA was clearly attenuated by bleomycin, but was recovered by EM703. EM703 also inhibited fibroblast proliferation and the collagen production in lung fibroblasts induced by Transforming growth factor-beta (TGF-β). The expression of Smad3 and Smad4 mRNA in murine lung fibroblasts disappeared due to TGF-β, but was recovered by EM703. EM703 inhibited the expression of p-Smad2/3 and Smad4 protein in murine lung fibroblasts induced by TGF-β.

**Conclusion:**

These findings suggest that EM703 improves bleomycin-induced pulmonary fibrosis in mice by actions of anti-inflammation and regulation of TGF-β signaling in lung fibroblasts.

## Background

Idiopathic pulmonary fibrosis (IPF) is a devastating disease with a five-year survival rate of less than 50% [1,2]. No treatments currently available improve the survival rate of patients with IPF, and novel therapeutic strategies are required.

Macrolides have been reported to improve the survival of patients with diffuse panbronchiolitis (DPB) and cystic fibrosis via anti-inflammatory effects [3,4]. We previously reported the preventive effects of 14-membered ring macrolides (14-MRMLs) in an animal experimental model of bleomycin-induced acute lung injury and subsequent fibrosis, which were mediated by anti-inflammatory mechanisms of action [5,6]. 

Recent publications have suggested novel treatment paradigms based on a more complete understanding of the pathogenesis of pulmonary fibrosis [7]. The development of pulmonary fibrosis is thought to include two phases: a persistent inflammatory phase and a sequential fibrotic phase [8]. Although the pathogenesis of pulmonary fibrosis remains unclear, many investigators have found that neutrophil-mediated lung injury occurring in the acute inflammatory phase plays an important role in the progression of interstitial pneumonia [9-11]. Fibroblast proliferation and extracellular matrix accumulation play a critical role in the subsequent fibrogenic process [1,12-14]. TGF-β plays a key role in the development of idiopathic pulmonary fibrosis [1,12-17] and in experimental animal models of pulmonary fibrosis [18-25], and TGF-β intercellular signaling from the cell membrane to the nucleus occurs through Smad proteins [26].

**Figure 1:**
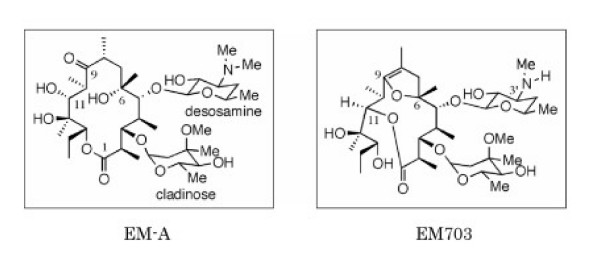
The structure of the erythromycin A (EM-A) and erythromycin 703 (EM703) was provided by the Kitasato Institute for Life Sciences at Kitasato University.

Macrolides have been reported to inhibit neutrophil-induced inflammation [3,5,6], and to inhibit the growth of nasal fibroblasts [27]. Bleomycin-induced lung injury and subsequent fibrosis in animals is a widely used experimental model of acute lung injury and fibrosis in humans [5,6,18-23,28-30]. EM703 is a new 12-membered ring macrolide derivative of erythromycin (Figure 1) prepared by the Kitasato Institute for Life Sciences in Kitasato University  without antibacterial effects [31]. It has recently been reported not only EM-A, but also EM703 suppressed the activation of nuclear factor (NF)-κB and the production of interleukin-8, demonstrating that the anti-inflammatory action of the macrolide is independent of its antibacterial activity [32]. We therefore investigated the effects of EM703 using an experimental model of bleomycin-induced acute lung inflammation and subsequent fibrosis in mice.

In this study, we found that EM703 has anti-inflammatory effects, as do 14-MRMLs, and found a new antifibrotic effect of EM703 in an experimental model of bleomycin-induced pulmonary fibrosis in mice. Our results suggest that the new antifibrotic effect of EM703 through the mechanisms of action of EM703 in the inhibition of Smad-mediated TGF-β signal transduction in murine lung fibroblasts.

## Materials and methods

### Mice and reagents

Seven-week-old male ICR mice (Nippon CLEA; Tokyo, Japan) weighing 30 g each on average were randomly assigned to groups. All experiments used eight mice/group, unless otherwise noted in the figure legends. Bleomycin (Nippon Kayaku; Tokyo, Japan) was dissolved in normal saline solution (NS) and administered intravenously to ICR mice at a dosageof 100 mg/kg body weight (0.3 ml per mouse). EM703 (Kitasato Institute for Life Sciences, Tokyo, Japan) at 75 mg/kg body weight was suspended in 5% gum arabic (AG) (Wako Pure Chemical Industries; Tokyo, Japan) at 0.3 ml per mouse and orally administered by force with a microtube daily to ICR mice.

### Schedule and evaluation of early-phase inflammation

NS was administered intravenously to the mice treated with NS alone (day 0). Bleomycin was administered intravenously to mice treated with bleomycin alone and bleomycin plus treatment with EM703 (day 0). AG was orally administered daily to mice of the NS-alone and bleomycin-alone groups from day -3 until the time of death. EM703 was administered daily to the EM703-treated groups from day -3 until the time of death. The mice in all groups were sacrificed under etheranesthesia on day 7 after bleomycin or NS injection. All groups were examined for cell populations in the BAL fluid and for induction of mRNA of Smad3 and Smad4 in the lung tissues by RT-PCR on day 7 after bleomycin or NS injection.

### Schedule and process of evaluation of late-phase fibrosis

The bleomycin-untreated groups included the NS-treated group [group 1] and the EM703-alone group [group 2]. The bleomycin-treated groups included bleomycin alone [group 3], bleomycin plus pretreatment with EM703 (day -3 to day 13) [group 4], and bleomycin plus post-treatment with EM703 (day 3 to day 20) [group 5]. NS was administered intravenously to the bleomycin-untreated mice (day 0). Bleomycin was administered intravenously to the bleomycin-treated mice (day0). AG was orally administered daily to the mice of groups 1 and 3 (day -3 to day 27). EM703 was orally administered dailyto the mice of group 2 (day -3 to day 27) and those of groups 4 (day -3 to day 13) and 5 (day 3 to day 20). The mice in all groups were sacrificed under etheranesthesia on day 28 after bleomycin or NS injection. Fibroticfoci were assessed histologically in the left lung tissues, and the hydroxyproline content in the right lung tissues was chemically determined.

### Histological analysis

For histological examination, 10% formalin-fixed lung tissues were embedded in paraffin. The paraffin sections were stained with either hematoxylin and eosin (HE) or Masson Trichrome (MT), and systematically scanned with a light microscope (OLYMPUS AX80, Tokyo, Japan). We compared the severity of interstitial fibrosis among the groups using the Ashcroft score [33].

### Hydroxyproline measurement

The total collagen content of the right lung was determined by hydroxyproline (HOP) assay [34]. After acid hydrolysis of the right lung with 12N HCL at 100°C for 20 hours in a sealed glass tube (Iwaki Tokyo, Japan), the HOP content was determined by high-performance liquid chromatography (HPLC).

### Analysis of BAL fluid

BAL fluid was obtained by the injection of 1 ml saline (three times, total 3 ml) followed by gentle aspiration of the fluid from the right and left lungs after securing an intratracheal catheter within a trachea. With this catheter, the ratios of the recovery of lavage fluid ranged from 70% to 80% and did not significantly differ among the groups. The total numbers of cells in the BAL fluid were counted with a hemocytometer. For differential counts of leukocytes in the BAL fluid, cytospin smear slides were prepared (Labsystems Japan) and stained with Giemsa solution (Merck Japan). Differential cell counts were performed on 200 cells per smear.

### Cell cultures

A murine lung fibroblast cell line, MLg2908 (ATCC, CCL-206), originating from ddY mice was maintained in Roswell Park Memorial Institute (RPMI1640, Immuno-Biological Laboratories Gunma, Japan) with 10% fetal calf serum (FCS, Eguitech-BIO, INC. Kerrville, TX). Cultures of it were grown in a 5% CO_2 _humidified atmosphere at 37°C. The cell groups tested included those of group 1 (control), group 2 [presence of TGF-β (TGF-β_1_, BD Biosciences, Bedford, MA) alone], and group 3 (presence of TGF-β and treatment with EM703).

### Assay of proliferation of murine lung fibroblast cell line

Lung fibroblast cells were suspended at 5 × 10^4^/ml in RPMI1640 with 10% FCS and plated in 96-well plates at 100 μl per well in a 5% CO_2 _humidified atmosphere at 37°C for incubation for 24 hr. The medium was changed to serum-free Dulbecco's Modified Eagle's Medium (DMEM, GIBCO™, Grand Island, NY, USA) in all groups, EM703 was added at various final concentrations for group 3 incubation for 24 hr. Thereafter, TGF-β was added at various final concentrations for group 2 and 3 incubation for 24 hr again. Each group's cells were incubated with a Cell Counting Kit-8 (DOJINDO; Tokyo, Japan) at 37°C for 3 hr. OD (450 nm) values were measured on a microplate reader (BIO-RAD Model 3550, Tokyo, Japan).

### Assay of soluble collagen production by lung fibroblast cell line

Lung fibroblast cells were suspended at 5 × 10^4^/ml in RPMI1640 with 10% FCS and plated in 24-well plates at 1 ml per well and incubated in a 5% CO_2 _humidified atmosphere at 37°C. After 24 hr of incubation, the medium was changed to serum-free DMEM in all groups, and EM703 (5 μg/ml) was added for group 3 incubation for 24 hr. Thereafter, TGF-β (5 ng/ml) was added for group 2 and 3, followed by incubation for 24 hr again. The cells of each group were then washed once and resuspended to 1 × 10^5 ^cells/ml in serum-free DMEM and plated in 24-well plates at 1 ml per well for incubation. After 24 hr of incubation, the supernatants were collected and measured for collagen concentration with a soluble collagen assay kit (Biocolor Ltd., UK).

### Cell cultures for the expression of Smad3 and Smad4 mRNA and protein assay

The cells of group 3 were divided into three subgroups as follows: group 3a: presence of TGF-β and pre-treatment with EM703; group 3b: presence of TGF-βand syn-treatment with EM703; and group 3c: presence of TGF-β and post-treatment with EM703. Lung fibroblast cells were suspended at 2 × 10^4^/ml in RPMI1640 with 10% FCS and plated in 24-well plates at 1 ml per well for incubation in a 5% CO_2 _humidified atmosphere at 37°C. After 48-hr of incubation, the medium was changed to serum-free DMEM, and EM703 (5 μg/ml) was added for group 3a, with incubation continued for 24 hr. Thereafter, TGF-β (5 ng/ml) was added to the cells of groups 2 and 3 (a, b, c). EM703 (5 μg/ml) was simultaneously added to the cells of group 3b. After 24 hr of incubation, EM703 (5 μg/ml) was added for group 3c, followed by incubation for an additional 24 hr. Each cell culture was examined for the expression of mRNA of Smad3 and Smad4 by RT-PCR and for expression of Smad3 and Smad4 protein assay by western blotting.

### Cell cultures for the expression of p-Smad2/3 protein assay

The cell groups tested included those of the control, the presence of TGF-β alone, and the presence of TGF-β and pre-treatment with EM703. Conducting the cell cultures and treatment with EM703 before the presence of TGF-β used the same method as the Smad3 and Smad4 protein assay in group 3a. The cells were cultured in the presence of TGF-β (5 ng/ml) for 15 min and 12 hr. Followed by the presence of TGF-β, the cells were collected and the expression of p-Smad2/3 protein was examined by western blotting.

### RT-PCR

Total RNA was extracted from each specimen of lung tissue (*in vivo*) and lung fibroblast cells (*in vitro*) using ISOGEN (Nippon GENE; Tokyo, Japan). The methods of RNA extraction and RT-PCR used have been previously described [6,35]. For the amplification of the desired cDNA, the following gene-specific primers were used[36,37]. Glucose-6-phosphate dehydrogenase(G6PD) was measured as an internal control [38].

Smad3: sense 5'-CTGGCTACCTGAGTGAAGATGGAGA-3',

antisense 5'-AAAGACCTCCCCTCCGATGTAGTAG-3'

Smad4: sense 5'-GTATAATGCCACCAGTACCACCAAC-3',

antisense 5'-TGACCCAAGCAAAAGCGATCTCCTC -3'

G6PD: sense 5'-TAGGAATTCATCATCATGGGTGCATCG-3'

antisense 5'-TAGAAGCTTGTTTGCGGATGTCAGCCACTGT-3'

The PCR products were electrophoresed on 2% agarose gels, stained with ethidium bromide, and observed with ultraviolet transillumination. Intensity analysis of the bands was performed with Adobe Photoshop 6.0 (Adobe Systems; Tokyo, Japan), with the expression of Smad3 mRNA indicated by the number of white pixel areas.

### Western blotting analysis

Western blotting was used for the measurement of Smad3, Smad4 and p-Smad2/3 protein assay protein in lung fibroblast cell line MLg2908. Total protein 100 μg was separated by 10% SDS-PAGE. Transfer to polyvinylidene difluoride membranes by a blot appliance (AE-6677P, ATTO CORPORATION, Tokyo, Japan) was performed according to the manufacturer's instructions. After having been blocked with 5% skim milk (Snow Brand Milk Production, Japan), the membrane was incubated with anti-Smad3 antibody (sc-8332, Santa Cruz Biotechnology, Inc. USA; rabbit polyclonal antibody, dilution 1/200) for 1 hr. The membrane was washed, and primary antibody was detected using alkaline phosphatase-conjugated affinipure goat anti-rabbit IgG (Jackson ImmunoResearch Laboratories, Inc. USA; dilution 1/10000) incubated for 1 hr. After having washed the membrane, the Smad3 protein band was visualized using an alkaline phosphatase substrate. Smad4 protein was detected by anti-Smad4 antibody (sc-7966, Santa Cruz Biotechnology, Inc. USA; mouse monoclonal antibody, dilution 1/100), and primary antibody was detected using alkaline phosphatase-conjugated affinipure goat anti-mouse IgG+IgM (Jackson ImmunoResearch Laboratories, Inc. USA; dilution 1/10000). Smad4 protein was detected using the same method as Smad3. p-Smad2/3 protein was detected by anti-p-Smad2/3 (sc-11769, Santa Cruz Biotechnology, Inc. USA; goat polyclonal antibody, dilution 1/100), and primary antibody was detected using donkey anti-goat IgG horseradish peroxidase (HRP) (sc-2056, Santa Cruz Biotechnology, Inc. USA; dilution 1/5000). P-Smad2/3 protein was detected by the chemiluminescence system (Amersham Biosciences ECL plus Western Blotting Detection System).

### Statistical analysis

Statistical analysis of the data was performed using Stat Mate III software (ATMS DIGITALS Medical Station, Tokyo, Japan). Comparisons between groups were performed using one-way ANOVA followed by the Newman-Keuls test. *P *values of less than 0.05 were considered significant.

## Results

### Changes in cell number in BAL fluid

**Figure 2:**
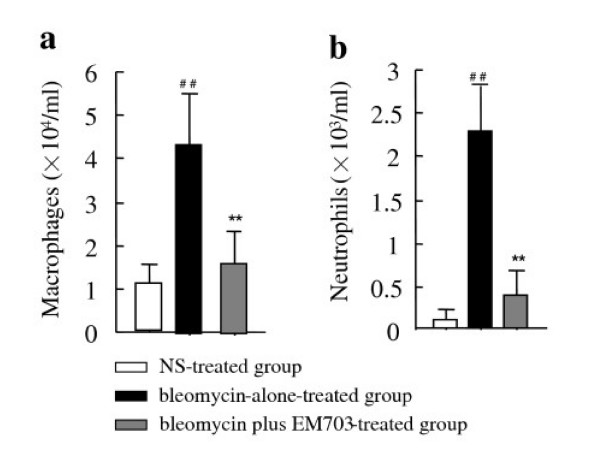
The number of macrophages **(a) **and neutrophils **(b) **in BAL fluid on day 7 after bleomycin injection in ICR mice (n = 8). ##*P *< 0.01, significantly different from NS-treated group; **P < 0.01, significantly different from bleomycin-alone-treated group. The values are means, and the bars are SD.

Numbersof macrophages and neutrophils in BAL fluid were significantly increased on day 7 after bleomycin injection. The increases in number of macrophages and neutrophils in BAL fluid were significantly attenuated by EM703 (Figure 2).

### Histopathologic assessment

**Figure 3:**
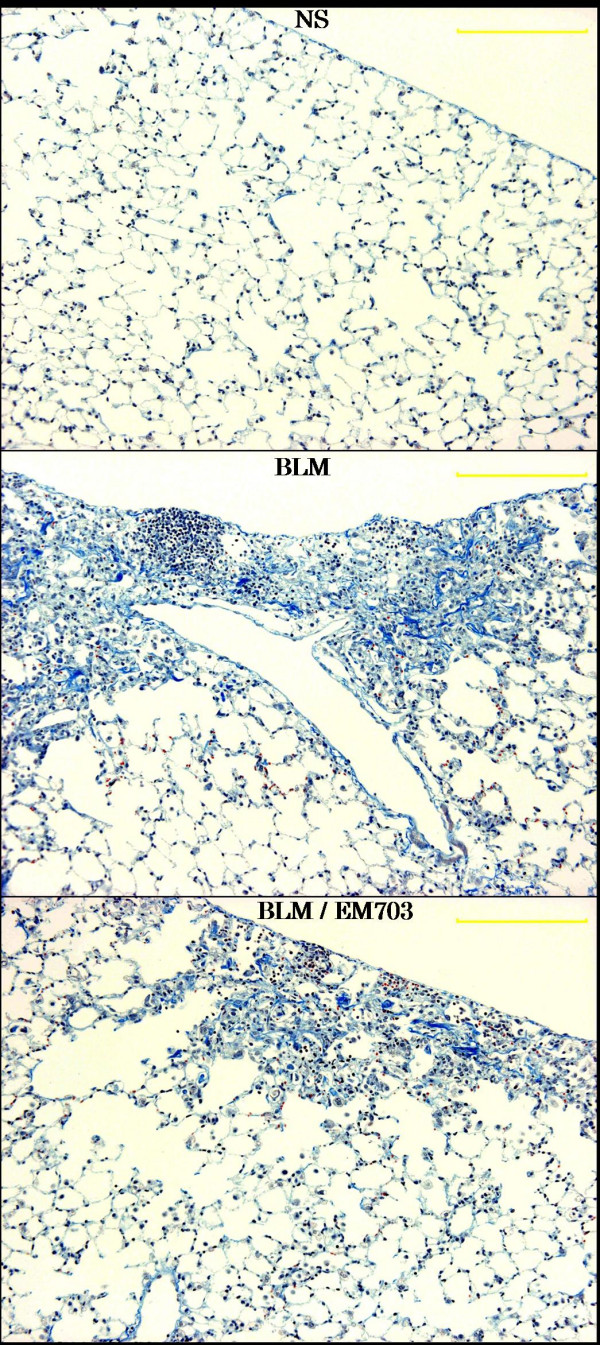
Pathologic features of lung tissues (Masson Trichrome stain) on day 28 after bleomycin injection in ICR mice. These photographs show typical results. NS: NS-treated group; BLM: bleomycin-alone-treated group; BLM/EM703: bleomycin-plus-EM703-treated group. The scale is 200 μm.

**Figure 4:**
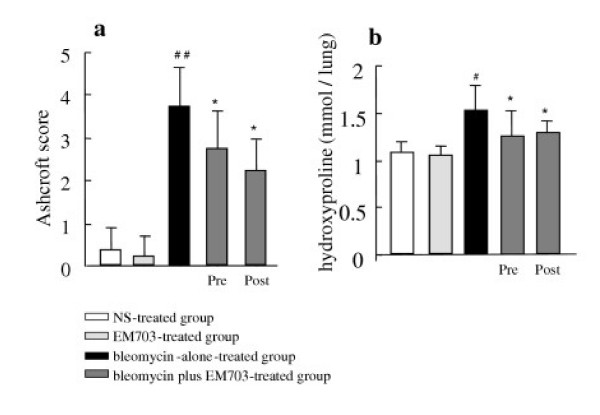
Histopathologic assessment of pulmonary fibrosis on day 28 after bleomycin injection in ICR mice by the Ashcroft score (n = 8) **(a)**. Comparison of hydroxyproline contents of lung tissues on day 28 after bleomycin injection in ICR mice (n = 8) **(b)**. pre: EM703-pretreated group (day – 3 to day 13); post: EM703-post-treated group (day 3 to day 20). #*P *< 0.05, ##*P *< 0.01, significantly different from NS-treated group; *P < 0.05, significantly different from bleomycin-alone-treated group. The values are means, and the bars are SD.

Bleomycin-induced pulmonary fibrosis was significantly inhibited by treatment with EM703 on day 28 after bleomycin injection in ICRmice. A typical picture of the attenuation of fibrosis is shown in Figure 3. Of the groups treated with EM703, the Ashcroft scores were significantly reduced compared to those in the bleomycin-alone group (Figure 4a). The administration of EM703 alone resulted in no remarkable changes in the results of histopathologic assessment of lung tissue.

### Hydroxyproline content in lung tissue

The concentration of hydroxyproline on day 28 after bleomycin injection was significantly higher in the bleomycin-alone group than in the NS-alone group. Of the groups treated with EM703, the hydroxyproline content was significantly reduced compared to that in the bleomycin-alone group. The administration of EM703 alone resulted in no remarkable changes in the hydroxyproline content of the lung tissue (Figure 4b).

### Assay of proliferation of MLg2908

**Figure 5:**
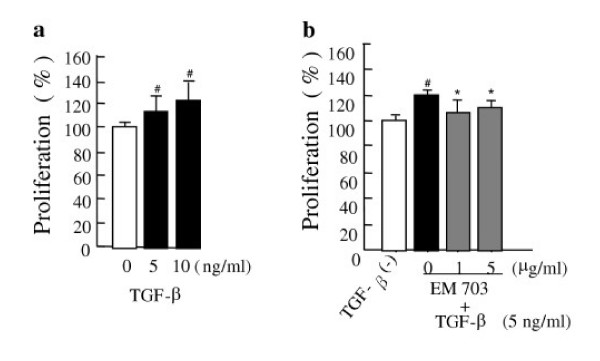
Effect of TGF-β on the proliferation of MLg 2908 **(a)**, and effect of EM703 on the proliferation of MLg 2908 induced by TGF-β **(b)**. #*P *< 0.05, significantly different from without-TGF-β group; *P < 0.05, significantly different from only-TGF-β-presence group. The results are expressed as mean ± SD of 8 replicate wells.

TGF-β significantly increased MLg2908 proliferation (Figure 5a). The proliferation of MLg2908 induced by TGF-β was significantly inhibited by EM703 (Figure 5b).

### Assay of soluble collagen production by MLg2908

**Figure 6:**
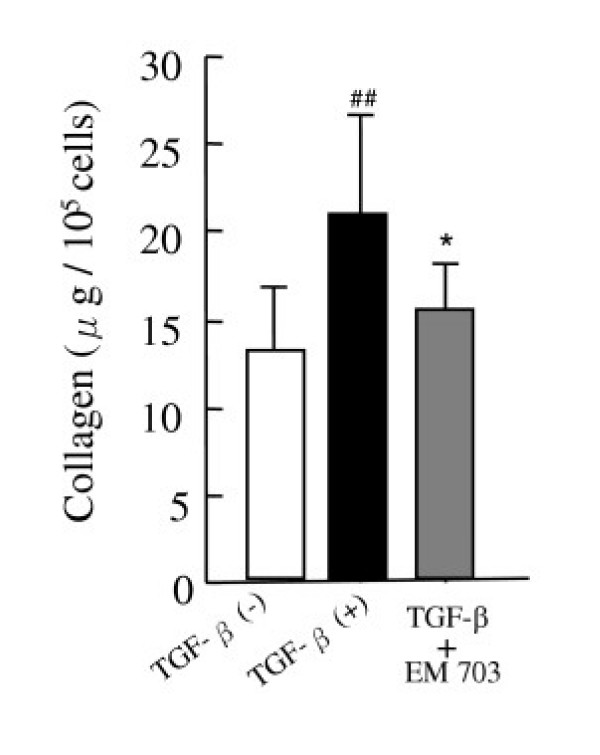
Effects of EM703 on the collagen production of MLg2908 induced by TGF-β. TGF-β: 5 ng/ml, EM703: 5 μg/ml. ##*P *< 0.01, significantly different from without-TGF-β group; *P < 0.05, significantly different from only-TGF-β-presence group. The results are expressed as the mean ± SD of 8 replicate wells.

TGF-β significantly increased the production of soluble collagen by MLg2908 cells. The increase in the measured concentration of soluble collagen induced by TGF-β was significantly inhibited by EM703 (Figure 6).

### Expression of Smad3 and Smad4 mRNA in lung tissues

**Figure 7:**
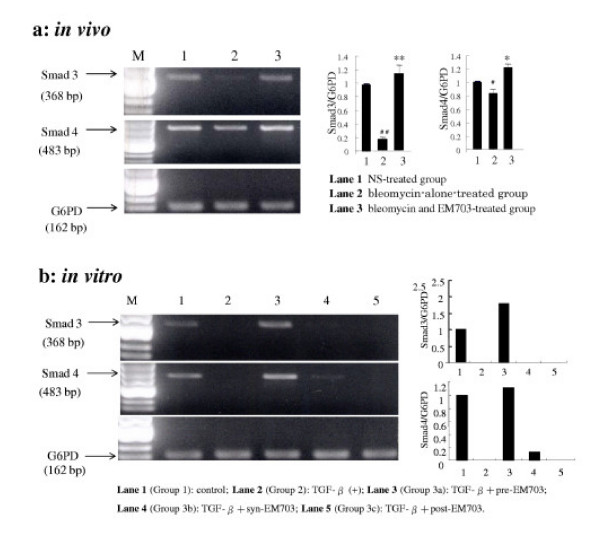
These photographs show typical results. Each density of PCR products was measured using Adobe Photoshop 6.0. G6PD was measured as an internal control. The ratio of each Smad molecule against G6PD is shown by a histogram. bp: base pair. **(a) **Effects of EM703 on the expression of Smad3 and Smad4 mRNA in lung tissue on day 7 after bleomycin injection in ICR mice (n = 3). #*P *< 0.05, ##*P *< 0.01, significantly different from NS-treated group; **P < 0.01, *P < 0.05, significantly different from bleomycin-alone-treated group. The values are means, and the bars are SD. (b) Expression of Smad3 and Smad4 mRNA in MLg2908. TGF-β: 5 ng/ml, EM703: 5 μg/ml. Each group as described in Materials and Methods: Cell cultures for the expression of mRNA and protein assay.

The expression of Smad3 mRNA was eliminated by bleomycin, but recovered to control level by treatment with EM703 on day 7 after bleomycin injection. The expression of Smad4 mRNA was attenuated by bleomycin, but recovered to a higher control level by treatment with EM703 on day 7 after bleomycin injection (Figure 7a).

### Expression of Smad3 and Smad4 mRNA in MLg2908

The expression of Smad3 and Smad4 mRNA was completely eliminated by the addition of TGF-β. The elimination of the expression of Smad3 and Smad4 mRNA by TGF-β was reversed to higher than the control level by pre-treatment with EM703, but was not recovered by syn-treatment or post-treatment with EM703 (Figure 7b).

### Expression of Smad3, Smad4 and p-Smad2/3 protein in MLg2908

**Figure 8:**
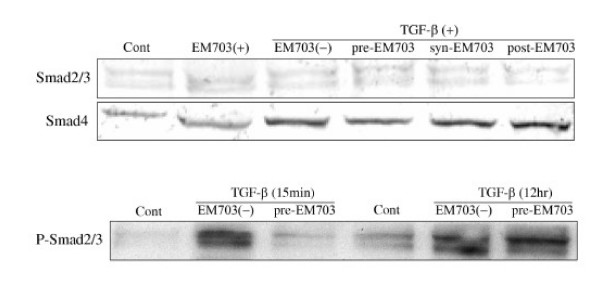
Expression of Smad3, Smad4 and p-Smad2/3 protein in MLg2908 by western blotting. TGF-β: 5 ng/ml, EM703: 5 μg/ml. Each group as described in Materials and Methods: Cell cultures for the expression of mRNA and protein assay.

The expression of Smad3 protein in murine lung fibroblasts was not changed by TGF-β. The expression of p-Smad2/3 protein was increased by TGF-β. The increased expression of p-Smad2/3 protein by TGF-β exposure for 15 min was remarkably inhibited by pre-treatment with EM703, but the increased expression of p-Smad2/3 protein by TGF-β exposure for 12 hr was not inhibited by pre-treatment with EM703. The expression of Smad4 protein was increased by TGF-β. The increased expression of Smad4 protein by TGF-β was inhibited by pre-treatment with EM703, but not inhibited by syn-treatment or post-treatment with EM703 (Figure 8).

## Discussion

We previously reported that 14-MRMLs inhibited the induction of vascular cell adhesion molecule 1 mRNA and leukocyte migration in the early inflammatoryphase, thereby preventing lung injury and fibrosis in bleomycin-challenged mice [6]. In the present study, we investigated the effects of EM703 – a new derivative of EM in the same experimental model in both the acute inflammatory phase and sequential fibrotic phase in mice.

Initially, to evaluate the effects of EM703 on the inflammatory phase, we investigated bleomycin-induced changes in the cell populations in BAL fluid on day 7 after bleomycin injection. The increase in the number of macrophages and neutrophils in the BAL fluidon day 7 after bleomycin injection was significantly attenuated by EM703 (Figure 2). Not only EM-A, but also EM703, suppressed the activation of NF-κB and the production of interleukin-8 [32]. Taken together, his finding suggests the possibility that EM703 also inhibits the migration of neutrophils and macrophages into the airspace, which would be an important anti-inflammatory mechanism in this model in addition to those possessed by 14-MRMLs [6].

To evaluate the effects of EM703 in the fibroticphase, we further investigated bleomycin-induced histopathologic changes and changes in hydroxyproline content in the lung tissues on day 28 after bleomycin injection, which is within the fibrotic phase. Bleomycin-induced pulmonary fibrosis on day 28 was significantly inhibited by treatment with EM703 (Figures 3, 4). The effectof EM703 on bleomycin-induced pulmonary fibrosis in mice appeared owing to the attenuation of inflammatory cell infiltration such as neutrophil and macrophage migration due to EM703, resulting in the inhibition of lung injury and subsequent fibrosis. This may be a mechanism of the antifibrotic effects of EM703.

In a previous study, the effectiveness of pre-treatment with 14-MRMLs (beginning 3 days before bleomycin injection to 13 days after bleomycin injection) was significantly stronger than that of post-treatment (beginning 3 days to 13 days after bleomycin injection)with 14-MRMLs [6]. In this study, the effectiveness of post-treatment with EM703 (beginning 3 days to 20 days after bleomycin injection) was almost equal to that of pre-treatment with EM703 (beginning 3 days before bleomycin injection to 13 days after bleomycin injection) (Figures 4). Practically, the numbers of macrophages and neutrophils returned to control levels at13 days after bleomycin injection [6]. The post-treatment with EM703 also significantly inhibited bleomycin-induced pulmonary fibrosis, suggesting that the mechanisms of action of EM703 against bleomycin-induced pulmonary fibrosis in mice may involve not only anti-inflammatory effects but also anti-fibrotic effects resulting in the direct attenuation of fibroblast proliferation.

It has been reported that fibroblast proliferation and extracellular matrix accumulation play an important role in the fibrogenic process [1,12-14]. TGF-β plays a key role in the development of idiopathic pulmonary fibrosis [1,12-17] and animal experimental modelsof lung fibrosis [18-25]. Our recent report suggests that the TGF-β level was increased in the same bleomycin-challenged mouse lung fibrosis model as this study [39]. To determine the mechanisms by which EM703 inhibits bleomycin-induced pulmonary fibrosis in mice, we further examined the effects of EM703 on the proliferation of and collagen production due to murine lung fibroblasts induced by TGF-β *in vitro*. Our findings indicated that the proliferation of murine MLg2908 lung fibroblasts induced by TGF-β was significantly inhibited by EM703 (Figure 5), and that the increase in the production of soluble collagen by TGF-β was significantly inhibited by EM703 (Figure 6). The mechanisms of inhibition by EM703 of bleomycin-induced pulmonary fibrosis in mice may involve the inhibition of TGF-β signaling, mediating fibroblast proliferation and extracellular matrix production.

TGF-β signaling from the cell membrane to the nucleus occurs via Smad proteins [26]. Smad2 and Smad3 are structurally highly similar and mediate TGF-β signals. Smad4 is distantly related to Smad2 and Smad3, and forms a heteromeric complex with Smad2 after TGF-β or activin stimulation. TGF-β induces heteromeric complexes of Smad2, 3 and 4, and their concomitant translocation to the nucleus, which is required for efficient TGF-β signal transduction [40]. Smad3 contributes to bleomycin-induced lung injury [41], and is a major component of the signal transduction pathway leading to fibrogenesis [42,43]. It has been reported that the expression of Smad3 mRNA was down-regulated at an early stage of inflammatory injury during bleomycin-induced pulmonary fibrosis, and the expression of Smad2 mRNA remained unchanged after bleomycin administration [44].

The most common theory of the pathogenesis of idiopathicpulmonary fibrosis is that the disease process begins with an 'alveolitis,' characterized by the accumulation of inflammatory cells. Neutrophils and mononuclear cells accumulate, and concomitant cytokines (like TGF-β) are released to stimulate fibroblast proliferation. Fibroblasts then migrate into areas of acute lung injury and are stimulated to secrete collagen and other matrix proteins [[1,8] and [12]].

Therefore, we examined the expression of Smad3 and Smad4 in lung tissue on early-phase day 7 after bleomycin injection. The results obtained were consistent with the reported data [44], that is, the expression of Smad3 mRNA was down-regulated at an early stage of inflammatory injury during bleomycin-induced pulmonary fibrosis (Figure 7a, Lane 2). The Smad4 mRNA was also down-regulated by bleomycin in this model (Figure 7a, Lane 2). The decrease in the expression of Smad3 and Smad4 mRNA by bleomycin was reversed to control level or higher than the control level by treatment with EM703 on day 7 after bleomycin injection (Figure 7a, Lane 3).

We further examined the regulation of the expression ofSmad3 and Smad4 mRNA by TGF-β in murine lung fibroblasts *in vitro*. The results showed that the expression of Smad3 and Smad4 mRNA was completely eliminated by TGF-β (Figure 7b, Lane 2). It has been demonstrated that there occurs an immediate translocation of Smad3 protein from the cytoplasm to the nucleus and a delayed down-regulation of Smad3 mRNA by TGF-β in lung fibroblasts [44]. Our results showed that the elimination of the expression of Smad3 and Smad4 mRNA by TGF-β (Figure 7b, Lane 2) was reversed to higher than the control level by pre-treatment with EM703 (Figure 7b, Lane 3), but was not recovered by syn-treatment (Figure 7b, Lane 4) or post-treatment (Figure 7b, Lane 5) with EM703.

The expression of Smad3 protein in murine lung fibroblasts was not changed by TGF-β. The expression of p-Smad2/3 and Smad4 proteins was remarkably increased by TGF-β. The increased expression of Smad4 protein was remarkably inhibited by pre-treatment with EM703, but was not inhibited by syn-treatment or post-treatment with EM703. The increased expression of p-Smad2/3 by TGF-β exposure for 15 min was remarkably inhibited by EM703, but the increased expression of p-Smad2/3 by TGF-β exposure for 12 hr was not inhibited by EM703 (Figure 8).

Both TGF-β receptor type I and type II are indispensable for TGF-β signaling [45-47]. We thus considered the possibility that the mechanisms by which EM703 inhibits TGF-β signal transduction in fibroblasts involve TGF-β receptors.

Recent work indicates that fibroblasts respond to TGF-β independently of Smad2/3 phosphorylation, and non-Smad TGF-β signaling pathways are also quite active in the bleomycin fibrosis models [48]. These are the results of studies mainly investigating the action of TGF-β_2_. TGF-β_1_, -β_2_, and -β_3 _are differentially expressed during bleomycin-induced lung fibrosis [23]. In this study, we investigated the effects of EM703 on the action of TGF-β_1 _in the murin lung fibroblasts. TGF-β_1 _plays a key role in the pathogenesis of pulmonary fibrosis, and the Smad3 pathway is involved in fibrogenesis [41-44].

Many investigators have found that fibroblasts migrate into areas of acute lung injury [8], in which fibroblastic foci represent an active form of fibroblasts [12]. TGF-β participates not only in the late phase but also the active early phase of acute lung injury [49-51]. The down-regulation of Smad3 in the early stage of inflammation and during the reparative phase was in contrast to the expression of collagen [44]. In this study, since the decrease in the expression ofSmad3 mRNA by bleomycin was reversed to higher than the control level by treatment with EM703 on day 7 after bleomycin injection, we emphasize that the antifibrotic effects of EM703 will be exhibited both in early inflammatory phase and more effectively in the reparative phase (Figure 4).

At present, there are no proven treatments for idiopathic pulmonary fibrosis. New strategies for such treatment have, however, been discussed, including the use of anti-inflammatory agents such as ONO5046 [52], antifibrotic agents such as pirfenidone [53,54] and immune modulators such as interferon gamma [55]. Many investigators have found that effective therapeutic strategies might include the modification of fibroblast replication, the modification of matrix deposition [1,14], the blocking of TGF-β [23,25,49], and the disruption of Smad3-mediated TGF-β signal transduction [41,42].

In this study, we found that EM703 improved bleomycin-induced pulmonary fibrosis in mice by inhibiting fibroblast TGF-β signal transduction, and clarified the anti-inflammatory and anti-fibrotic effects of EM703 in the attenuation of bleomycin-induced pulmonary fibrosis. Although there is a room for further investigation of the mechanism of EM703 inhibition of bleomycin-induced lung fibrosis, we believe that at least the anti-inflammation action and the signal control action of TGF-β will work. We found no deaths or abnormal reactions with a daily administration of 75 mg/kg body weight of EM703 during the experiments. Our results suggest that EM703 may be a promising new, safe agent for the treatment of pulmonary fibrosis, with both anti-inflammatory and anti-fibrotic effects.

## Authors' contributions

YL, AA, JU, SA and KM carried out the pathological studies, cell biology studies and molecular genetic studies. YL and AA participated in the design of the study, sequence alignment and drafted the manuscript. YL, KM, TS, YH and HI carried out the immunoassays. YL and TK performed the statistical analysis. TS, ST, SK, and SO, conceived of the study, and participated in its design and coordination. All authors read and approved the final manuscript.
